# Supplementary material for: Sperm sequencing reveals extensive positive selection in the male germline
Source: Nature. 2025 Oct 8;647(8089):421–8. doi: 10.1038/s41586-025-09448-3 (PMC12611766; doi:10.1038/s41586-025-09448-3)
Supplement: Supplementary file 2 — Reporting Summary [file 41586_2025_9448_MOESM2_ESM.pdf]

nature portfolio

Corresponding author(s): Raheleh Rahbari

Last updated by author(s): May 13, 2025

Reporting Summary

Nature Portfolio wishes to improve the reproducibility of the work that we publish. This form provides structure for consistency and transparency in reporting. For further information on Nature Portfolio policies, see our Editorial Policies and the Editorial Policy Checklist.

Statistics

For all statistical analyses, confirm that the following items are present in the figure legend, table legend, main text, or Methods section.

n/a	Confirmed	
☐	☒	The exact sample size (*n*) for each experimental group/condition, given as a discrete number and unit of measurement
☐	☒	A statement on whether measurements were taken from distinct samples or whether the same sample was measured repeatedly
☐	☒	The statistical test(s) used AND whether they are one- or two-sided<br>*Only common tests should be described solely by name; describe more complex techniques in the Methods section.*
☐	☒	A description of all covariates tested
☐	☒	A description of any assumptions or corrections, such as tests of normality and adjustment for multiple comparisons
☐	☒	A full description of the statistical parameters including central tendency (e.g. means) or other basic estimates (e.g. regression coefficient) AND variation (e.g. standard deviation) or associated estimates of uncertainty (e.g. confidence intervals)
☐	☒	For null hypothesis testing, the test statistic (e.g. *F*, *t*, *r*) with confidence intervals, effect sizes, degrees of freedom and *P* value noted<br>*Give P values as exact values whenever suitable.*
☒	☐	For Bayesian analysis, information on the choice of priors and Markov chain Monte Carlo settings
☒	☐	For hierarchical and complex designs, identification of the appropriate level for tests and full reporting of outcomes
☒	☐	Estimates of effect sizes (e.g. Cohen's *d*, Pearson's *r*), indicating how they were calculated

Our web collection on statistics for biologists contains articles on many of the points above.

Software and code

Policy information about availability of computer code

Data collection	None.
Data analysis	NanoSeq variant calling pipeline (https://github.com/cancerit/NanoSeq; v3.3 was used to process duplex sequencing data. R (v4.3.1) was used for statistical analysis and visualization. Code modified from trackViewer (R/Bioconductor package v1.38.0) was used to generate gene mutation "lollipop" plots. lme4 (v1.1-33) was used for linear mixed-effects models. ggplot2 (v3.4.4) was used for plotting.<br>dNdScv (https://github.com/im3sanger/dndscv; version as of commit on Sep 29, 2023) was used for selection analysis. HDP (https://github.com/nicolaroberts/hdp) was used for de novo mutational signature extraction. SigProfilerAssignment (v0.0.27; https://github.com/AlexandrovLab/SigProfilerAssignment) was used for COSMIC signature fitting.<br>All custom analysis code and scripts are available at https://github.com/mattnev17/spermPositiveSelectionManuscript.

For manuscripts utilizing custom algorithms or software that are central to the research but not yet described in published literature, software must be made available to editors and reviewers. We strongly encourage code deposition in a community repository (e.g. GitHub). See the Nature Portfolio guidelines for submitting code & software for further information.

Data

Policy information about availability of data

All manuscripts must include a data availability statement. This statement should provide the following information, where applicable:

- Accession codes, unique identifiers, or web links for publicly available datasets
- A description of any restrictions on data availability
- For clinical datasets or third party data, please ensure that the statement adheres to our policy

Raw sequencing data are available on the European Genome-Phenome Archive under accession number X. All non TwinsUK files necessary to recreate results are available at https://github.com/mattnev17/spermPositiveSelectionManuscript. Additional TwinsUK individual-level data are not permitted to be publicly shared or deposited due to the original consent given at the time of data collection, where access to these data is subject to governance oversight. All data access requests are overseen by the TwinsUK Resource Executive Committee (TREC). Requests will be reviewed within 4–6 weeks. For information on access to these genotype and phenotype data and how to apply, see https://twinsuk.ac.uk/researchers/access-data-and-samples/request-access/.
This study also made use of the following publicly available resources: the Developmental Disorder Genotype–Phenotype Database (DDG2P) at https://www.deciphergenomics.org/ddd/ddgenes, Online Mendelian Inheritance in Man (OMIM) at https://omim.org, the COSMIC Cancer Gene Census at https://cancer.sanger.ac.uk/census, gnomAD v2.1.1 at https://gnomad.broadinstitute.org, ClinVar at https://www.ncbi.nlm.nih.gov/clinvar/, and bisulfite methylation data from the ENCODE Project (testis samples ENCFF638QVP and ENCFF715DMX) available at https://www.encodeproject.org.

Research involving human participants, their data, or biological material

Policy information about studies with human participants or human data. See also policy information about sex, gender (identity/presentation), and sexual orientation and race, ethnicity and racism.

Reporting on sex and gender	All individuals self reported as male and provided a sperm sample.
Reporting on race, ethnicity, or other socially relevant groupings	All individuals that provided ethnicity information self-reported as "white".
Population characteristics	Sperm samples were collected at ages 24-75 years. Blood samples were collected at ages 22-83. Sample count, timepoint, and twin relationships are summarized in Supplementary Table 1.
Recruitment	Participants were recruited through the TwinsUK registry, a volunteer cohort of adult twins in the UK. Inclusion in this study was based on availability/willingness to donate high-quality semen and blood samples from male participants, as well as consent for genetic research. As a volunteer-based cohort, there is potential for self-selection bias, including overrepresentation of individuals who are more health-conscious or engaged with research. While this may limit generalizability to the broader population, the biological processes under study—mutation accumulation and selection in spermatogenesis—are unlikely to be strongly influenced by these factors. We also note that no individuals in the cohort had known genetic disorders or exposures (e.g. chemotherapy) likely to affect germline mutation patterns.
Ethics oversight	This study was carried out under TwinsUK BioBank ethics, approved by North West – Liverpool Central Research Ethics Committee (REC reference 19/NW/0187), IRAS ID 258513 and earlier approvals granted to TwinsUK by the St Thomas' Hospital Research Ethics Committee, later London – Westminster Research Ethics Committee (REC reference EC04/015).

Note that full information on the approval of the study protocol must also be provided in the manuscript.

Field-specific reporting

Please select the one below that is the best fit for your research. If you are not sure, read the appropriate sections before making your selection.

☒ Life sciences ☐ Behavioural & social sciences ☐ Ecological, evolutionary & environmental sciences

For a reference copy of the document with all sections, see nature.com/documents/nr-reporting-summary-flat.pdf

Life sciences study design

All studies must disclose on these points even when the disclosure is negative.

Sample size	The number of samples was determined by availability from the TwinsUK cohort.
Data exclusions	Some samples were excluded based on failed sequencing (Supplementary Table 1). Six sperm samples were excluded based on sperm count <1M/ml, which was not predetermined but based off of analyses in Supplementary Note 1. Three blood samples were excluded based off of a predetermined contamination threshold of verifyBamID alpha value above 0.005 (Abascal et al, 2021). Three sperm samples were excluded based off of a verifyBamID alpha value above 0.002, which was determined by analyses in the paper showing sperm needed a more stringent threshold than blood.
Replication	The mutation burdens described in sperm and blood were verified by comparing to well known estimates of these values from previous

Replication	publications. Analyses of positive selection replicated 9 of 13 known genes that drive this effect but otherwise describes the landscape of mutations using all available sample material.
Randomization	Randomization was not relevant to this study because it is an observational analysis of naturally occurring mutations in human sperm and blood samples. No interventions or group assignments were made, and sample comparisons (e.g., by age) reflect inherent biological variation rather than experimental manipulation.
Blinding	Blinding was not relevant to this study because it involved computational analysis of sequencing data without subjective measurements or investigator-assigned interventions. All variant calling, mutation burden estimation, and statistical analyses were performed using predefined pipelines or automated methods, minimizing risk of bias.

Reporting for specific materials, systems and methods

We require information from authors about some types of materials, experimental systems and methods used in many studies. Here, indicate whether each material, system or method listed is relevant to your study. If you are not sure if a list item applies to your research, read the appropriate section before selecting a response.

Materials & experimental systems

n/a	Involved in the study	
☒	☐	Antibodies
☒	☐	Eukaryotic cell lines
☒	☐	Palaeontology and archaeology
☒	☐	Animals and other organisms
☒	☐	Clinical data
☒	☐	Dual use research of concern
☒	☐	Plants

Methods

n/a	Involved in the study	
☒	☐	ChIP-seq
☒	☐	Flow cytometry
☒	☐	MRI-based neuroimaging

Plants

Seed stocks	*Report on the source of all seed stocks or other plant material used. If applicable, state the seed stock centre and catalogue number. If plant specimens were collected from the field, describe the collection location, date and sampling procedures.*
Novel plant genotypes	*Describe the methods by which all novel plant genotypes were produced. This includes those generated by transgenic approaches, gene editing, chemical/radiation-based mutagenesis and hybridization. For transgenic lines, describe the transformation method, the number of independent lines analyzed and the generation upon which experiments were performed. For gene-edited lines, describe the editor used, the endogenous sequence targeted for editing, the targeting guide RNA sequence (if applicable) and how the editor was applied.*
Authentication	*Describe any authentication procedures for each seed stock used or novel genotype generated. Describe any experiments used to assess the effect of a mutation and, where applicable, how potential secondary effects (e.g. second site T-DNA insertions, mosiacism, off-target gene editing) were examined.*
